# Proximity to Heavy Traffic Roads and Patient Characteristics of Late of Onset Asthma in an Urban Asthma Center

**DOI:** 10.3389/fmed.2021.783720

**Published:** 2021-12-16

**Authors:** Ting-Yu Lin, Horng-Chyuan Lin, Yun-Sheng Liu, Yu-Lun Lo, Chun-Hua Wang, Po-Jui Chang, Chun-Yu Lo, Shu-Min Lin

**Affiliations:** ^1^Department of Thoracic Medicine, Chang Gung Memorial Hospital, Taipei, Taiwan; ^2^College of Medicine, Chang Gung University, Taoyuan, Taiwan; ^3^BalDr Strategic Consulting (Hong Kong) Ltd., Taipei, Taiwan

**Keywords:** late onset asthma, traffic proximity, urban environment, asthma phenotype, traffic density

## Abstract

**Background:** Traffic-related pollution is associated with the onset of asthma and the development of different phenotypes of asthma. Few studies have investigated the association between traffic proximity and late-onset of asthma (LOA) and early-onset asthma (EOA). This study was conducted to investigate the associations of LOA phenotypes with a function of the distance between residence and heavy traffic roads (HTRs).

**Methods:** The study group consisted of 280 patients who were (LOA: 78.4%) recruited consecutively from a pay-for-performance asthma program to clarify the patient characteristics and proximity to HTRs within 1,000 m from their residences between EOA and LOA in three urban centers in Taiwan. The subsequent analysis focused on patients with LOA (*n* = 210) linking phenotypes and distance to HTRs.

**Results:** Subjects with LOA tended to be older than those with EOA and had shorter asthma duration, poorer lung function, lower atopy, and less exposure to fumes or dust at home. Patients with LOA were more likely than those with EOA to live within 900 m of two or more HTRs (14.3 vs. 3.4%, *p* = 0.02). Among patients with LOA, minimum distance to an HTR was negatively associated with numbers of specific IgE as well as positively associated with the age of onset and body weight significantly. A higher proportion of patients with atopy (26.3 vs. 20.6%, *p* = 0.001. odds ratio [OR]: 2.82) and anxiety/depression (21.0 vs. 18.1%, *p* = 0.047. OR: 1.81) and a trend of lower proportion of patients with obese (5.7 vs. 12.4%, *p* = 0.075) were found to be living within 900 m from HTRs.

**Conclusions:** Late-onset of asthma (LOA) tended to live in areas of higher HTR density compared to EOAs. Among patients with LOA living close to HTRs, the interaction between traffic-related pollution, allergy sensitization, and mood status were the factors associated with asthma onset early. Obesity may be the factor for later onset who live far from HTRs.

## Background

After an initial appearance in childhood, asthma may remain inactive for an extended period or reappear later in life. A number of recent studies have also described the onset of asthma during adulthood (1). Thus, early-onset asthma (EOA) and late-onset asthma (LOA) can be viewed as two distinct phenotypes, based on the categorization of disease entities according to underlying mechanisms or endotypes, such as risk factors, remission rates, co-morbidities, and gene expression profiles (2–4). The age at diagnosis determining the early vs. late-onset asthma varies from 12 or 18 years (4–6) of age to 40 (7) years of age. However, emerging evidence indicates that environmental factors also play a critical role in the development of asthma. The risk factors of EOA include single nucleotide polymorphisms on chromosome 17q21, atopic status, rhinovirus infection, and exposure to traffic-related air pollution (TRAP) (8–12). TRAP is one of the major environmental impacts of urbanization and previous research has shown the long-term effects on asthma onset in children (5, 13). Among children, proximity to traffic has been linked to an elevated risk of asthma (12, 14–16).

Despite the non-negligible incidence of adult-onset asthma, the causes have not been extensively investigated. Identifying the risk factors of LOA is crucial to understanding the underlying mechanisms and LOA was identified as the most significant independent risk factor for non-remission in patients with asthma (17). It should be noted however that the risk factors for LOA are more complex than those for EOA. Specific characteristics have been mentioned for their different influence on EOA and LOA. A population-based study in Finland showed the incidence of allergic asthma decreases with advancing age and after the age of 40, new asthma cases are almost non-atopic (18). The other study in Finland discovered the influence of the family history of asthma is higher on EOA than LOA (risk ratio (RR): 4.10 vs. 1.44) (9). A European survey discovered patients with chronic rhinosinusitis reported less EOA (RR: 0.45) but more LOA (RR: 3.09) (19). In the cluster study of asthma phenotypes, obese female is one of the major cluster of LOA (20). One large-scale genome-wide association study (GWAS) suggested that non-genetic risk factors play a more important role in LOA than in EOA. This is a clear indication that environmental factors are worthy of further consideration (21). The important role of TRAP in adult-onset asthma has also been emphasized (22). The association between traffic proximity and LOA has been identified by a certain amount of traffic volume and patients in risk (23–25). One study of our adult asthma cohort reported that higher IL-17A expression in the epithelium of patients among those living within 1,000 m from heavy traffic roads (HTRs) than among those living more than 1,000 m from HTRs (26).

In the current study, we were interested in whether the traffic proximity was different in EOA and LOA. We sought to determine whether proximity or density of HTRs is associated with the LOA in the asthma cohort of an urban medical center. We also examined patient characteristics in order to identify factors significantly associated with traffic proximity in LOA.

## Materials and Methods

### Study Design and Subject Recruitment

This was a cross-sectional study of asthma patients recruited consecutively in a pay-for-performance program at Chang Gung Memorial Hospital, Linkou branch, which has been implemented by the National Health Insurance Administration (NHIA) in Taiwan since 2001 (27). Certified physicians and case managers provide in-person training pertaining to asthma control, asthma care planning, and proper inhaler usage. The outcomes are regularly monitored by the NHIA (https://www.nhi.gov.tw/Content_List.aspx?=nEBDEAEDEC639490C&topn=5FE8C9FEAE863B46). Inclusion criteria included the diagnosis of asthma by a pulmonologist in accordance with ICD-10 code J45 at least twice within 90 days. Note that diagnoses were based on episodic respiratory symptoms (wheezing, breathlessness, chest tightness, and cough), variable or persistent obstructive pulmonary function, and response to asthma therapy. All patients provided written informed consent. The study protocol was approved by the Chang Gung Medical Foundation Institutional Review Board (No. 201900211B0).

### Patient Data Collection

At the initial recruitment, we recorded the characteristics of the subjects based on questionnaires or medical records, including age at the time of asthma diagnosis by a physician, asthma control test (ACT) results, family history of asthma, the use of asthma medication, co-morbidities, childhood history of dyspnea, frequency of bronchitis, exposure to fumes or dust at home or work, smoking status, and current residence (in the last 6 months). Pulmonary function and allergy-related biomarkers, including eosinophils, eosinophil cation protein (ECP), immunoglobulin E (IgE), and specific IgE (ImmunoCAP, Phadia, Sweden) were recorded. Patients with any positive specific IgE to allergens (>0.35 KU/L,) were considered atopic. The tests above were done at physicians' discretion in real-world practice. For example, according to the regulation of Taiwan healthcare insurance, the reimbursement of specific IgE would not be offered unless the total IgE > 25 KU/L. Therefore, a test of specific IgE was not mandatory and the numbers of sIgE to check were based on the physicians' discretion.

### Outcome Measurement

The total cohort of 283 patients was divided into two groups according to age at the time of asthma onset. Patients who were ≥ 18 years old at the time of asthma onset without a childhood history of dyspnea and frequency of bronchitis were defined as LOA. Otherwise, they were considered as EOA. It was determined that 94.7% of the total cohort were living in major urban centers: EOA (*n* = 58) and LOA (*n* = 210). This group of urban patients was subjected to further analysis to determine the proximity to HTRs within 1,000 m from their residences (26, 28) and whether distance to HTRs affected the age of asthma onset (analysis plan was illustrated in Supplementary Figure S1).

### Definition of Heavy Traffic Roads (HTRs)

Heavy traffic roads (HTRs) were identified using open-data daily PCU (Passenger Car Unit) statistics from the Directorate General of Highways of Taiwan (https://www.thb.gov.tw/sites/en/). Based on the geographic distribution of patients, we selected the ten busiest traffic monitoring sites, each of which had a daily mean bidirectional PCU exceeding 36,329 in 2018 (Supplementary Figure S2).

### Model of Geometric Analysis

Geometric data were extracted from maps obtained from the open-data service – Open Street Map (https://www.openstreetmap.org/export). Data covered the region between latitudes from 24.74 to 25.33 N and longitudes from 120.86 to 122.04 E. All map data other than roads and streets were excluded. Criteria for the selection of routing vectors were based on the Top-10 PCU routes. We sought to mitigate sphere projection bias by re-projecting the coronadite system of geometrics (e.g., routes or patient locations) from EPSG:4326 (WGS84 – World Geodetic System 1984, used in GPS) to EPSG:32651 (WGS 84 – UTM Zone 51N). This converted the spherical representation in radian units into a 2D flat surface presentation in meters, which is a convenient format for subsequent calculation. Geodata was processed using a custom script written in JavaScript under the NodeJS (version 12.9.1. OpenJS Foundation. San Francisco, California. Joyent, Inc.) runtime environment, and all exchangeable data formats were standardized according to GeoJSON format. The graphical representation of input variables and calculation results was handled using QGIS software (version 3.10.1. QGIS.org, 2021. QGIS Geographic Information System. QGIS Association. https://www.qgis.org).

Linear algebra (i.e., point to vector distance) was used to calculate the minimum distance between the domicile of each patient and the nearest HTR based on geometric data (Supplementary Figure S3). We also calculated the overall density of traffic in the areas surrounding the domicile of each patient by counting the number of HTRs within circles of various sizes, starting at 100 m and extending to a maximum distance of 1,000 m (26, 28).

### Statistical Analysis

All data were expressed as mean ± *SD* or percentage. The Student's *t*-test was used to compare the means of continuous variables and normally distributed data; otherwise, the Mann-Whitney test was used. Categorical variables including patients with EOA and LOA living in ≥ 1 or 2 heavy-traffic roads within indicated distance were tested using the Chi-square test or Fisher exact test. For tests done by physicians' discretion, the numbers are tested were smaller than the total cohort. The analysis was done on the patients who were checked and the number of participants who provided information and the number of participants with positive results were specified. Unadjusted odds ratio (OR) and 95% CI were calculated for selective variables in geometric analysis during the Chi-square test. The association between the minimum distance to an HTR and patient factors was tested using the Spearman rank correlation because the distance was not normally distributed. All analysis was performed using IBM SPSS Statistics version 19. Armonk, NY: IBM Corp. Statistically significant results were defined as *p* ≤ 0.05.

## Results

### Characteristics of Patients With LOA Differed From Those of Patients With EOA

Between July 2019 and June 2020, a total of 283 asthma patients were consecutively enrolled in the pay-for-performance program. Among these asthmatics, 222 subjects (78.4%) were LOA. Table 1 shows the characteristics of patients with EOA and LOA. Supplementary Figure S4 presents the distribution of patient ages and ages of onset. The mean age of onset of EOA and LOA was 8.6 years and 52.2 years, respectively. Compared to the EOA group, participants with LOA were older, had a shorter duration of asthma onset, less association of asthma family history, less exposure to fumes or dust at home, and a higher proportion of smoking habits. There was no difference in gender, co-morbidities, weight status, exacerbation history in the last year, and ACT score between the two groups. The pre-bronchodilator forced vital capacity (FVC) and forced expiratory volume in one second (FEV_1_) were lower in patients with LOA than in patients with EOA. As for allergic biomarkers, IgE levels were significantly higher in the EOA group than in the LOA group. The proportion of patients with ECP levels exceeding the normal range (18 μg/L) was higher in the EOA group than in the LOA group. The two groups were comparable in terms of eosinophil count. Patients with EOA tended to be more atopic and were also more susceptible to home dust mite (HDM), cat dander, and dog dander than patients with LOA (Supplementary Table S1).

**Table 1:** The characteristics of patients with early and late-onset asthma.

**Variables**	**EOA**	**LOA**	***p* value**
	**(*n* = 61)**	**(*n* = 222)**	
Age, years, mean (SD)	46.6 (19.2)	60.9 (15.5)	<0.001
Male, *n* (%)	29 (47.5)	108 (48.6)	0.9
Body mass index, kg/m^2^	25.7 (4.9)	25.8 (5.0)	0.8
Never smoker	48 (81.4)	142 (64.3)	0.006
ACT score, mean (SD)	19.7 (5.1)	21.0 (4.3)	0.1
Age of asthma onset, years, mean (SD)	8.6 (5.4)	52.2 (17.9)	<0.001
Asthma duration, years, mean (SD)	38.0 (21.1)	9.0 (12.9)	<0.001
Family history of asthma, *n* (%)	26 (42.6)	48 (21.6)	0.02
Home exposure to fumes or dust, *n* (%)	28 (45.9)	71 (32.0)	0.049
Occupational exposure to fumes/dust, *n* (%)	25 (41.0)	76 (34.2)	0.4
Comorbidities, *n* (%)			
Gastroesophageal reflux	29 (47.5)	110 (49.5)	0.9
Allergic rhinitis	42 (68.9)	137 (61.7)	0.3
Rhinosinusitis with or without polyp	14 (23.0)	53 (23.9)	0.9
Aspirin sensitivity	5 (8.2)	15 (6.8)	0.7
Anxiety or depression	23 (37.7)	87 (39.2)	0.8
Obstructive sleep apnea	9 (14.8)	51 (23.0)	0.2
Pulmonary function and allergic status			
FVC, Liter	2.74 (1.12)	2.17 (0.87)	<0.001
FVC, % of pred.	82.5 (18.9)	74.2 (20.2)	0.005
FEV1, Liter	2.12 (0.98)	1.63 (0.71)	<0.001
FEV1, % of pred.	75.3 (22.0)	68.3 (21.2)	0.03
IgE level, KU/L, median (range)	209.0 (6–2,075)	159.5 (2–283)	0.04
ECP level, μg/L, mean (SD)	16.1 (13.4)	15.0 (25.7)	0.8
ECP level ≥ normal range (18μg/L)	40.4 (21/52)	17.9 (29/162)	0.02
Eosinophil counts, cells/μL, mean (SD)	204.9 (182.2)	215.8 (218.8)	0.8
Atopy, % (n/N)	69.1 (38/55)	47.3 (87/184)	0.05

*Data are presented as number and percentage or mean and SD, unless otherwise indicated*.

*N, number of participants who provided information; n, number of participants with positive results*.

*EOA, early-onset asthma; LOA, late-onset asthma; ACT, asthma control test; FVC, forced vital capacity; FEV1, forced expiratory volume in 1 s; BD; pred., prediction. IgE, immunoglobulin E; ECP, eosinophil cationic protein*.

### The Distribution of Patients With EOA and LOA Living in Areas of High Traffic Density

**Figure 1:**
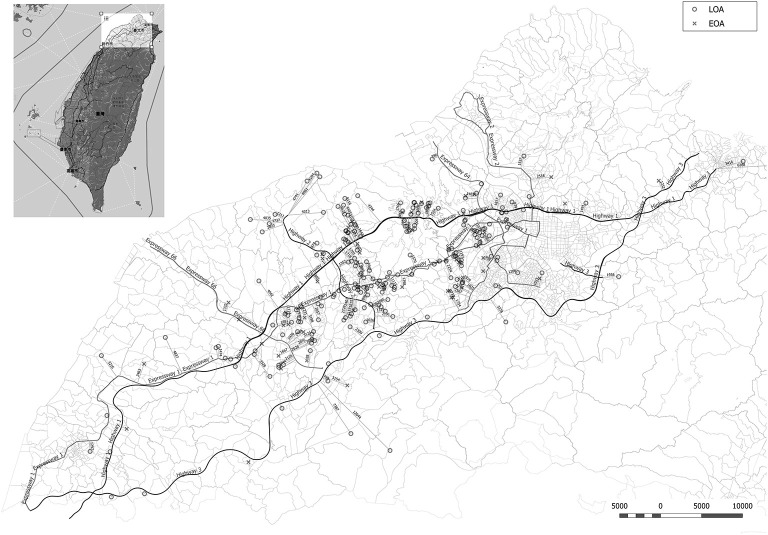
Minimum distance from the residence of asthmatic patients to the nearest heavy traffic road in New Taipei City, Taoyuan, and Hsinchu, Taiwan. EOA residences are indicated by X and LOA residences are indicated by circles. The heavy traffic roads in this study included three national highways (Highway 1, 2, and 3) and five expressways (Expressway 1, 2, 3, 64, and 66).

**Figure 2:**
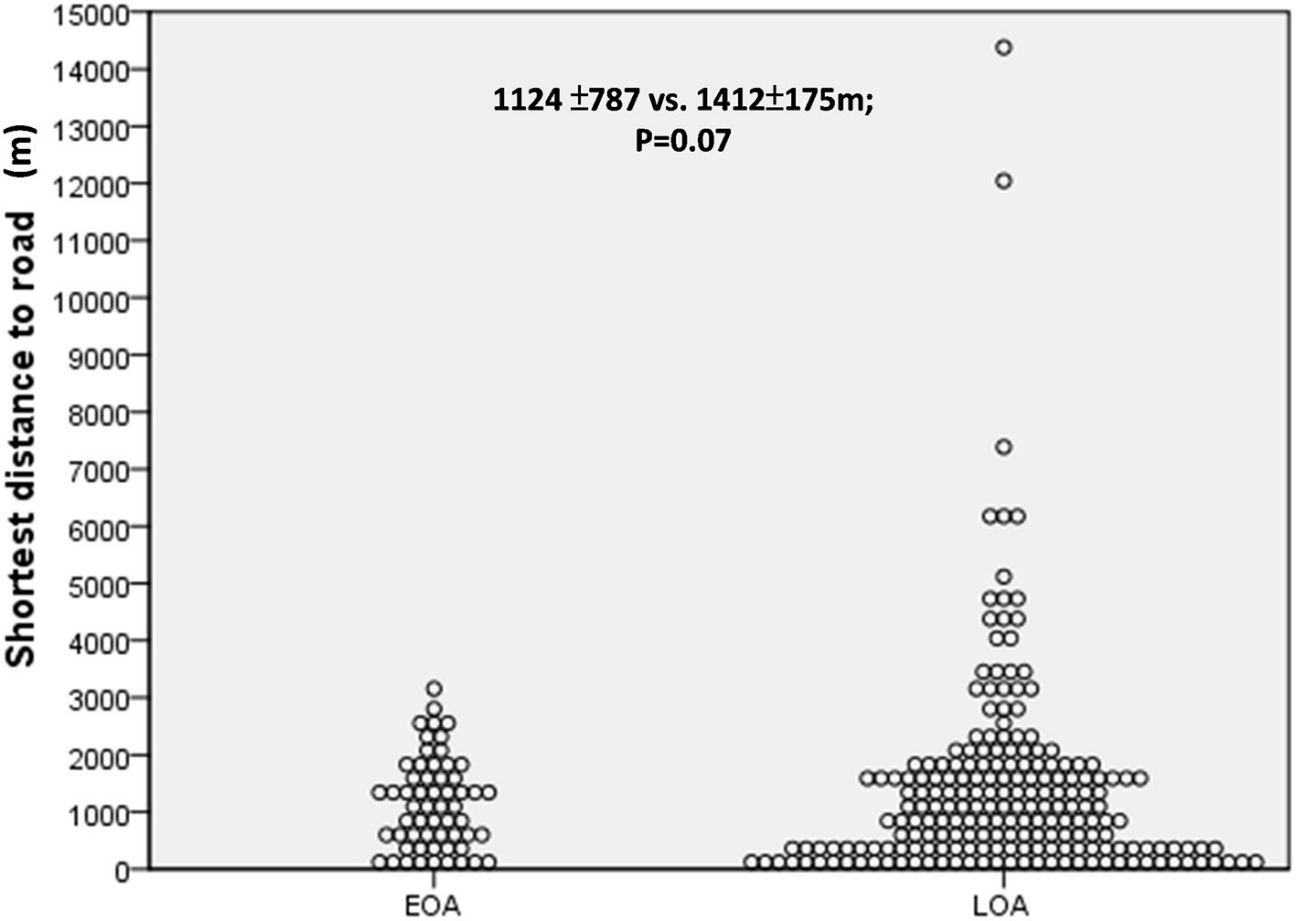
The minimum distance to the nearest heavy traffic road (HTR) between early-onset asthma (EOA) vs. late-onset asthma (LOA). No significant difference was observed between patients with EOA and LOA in terms of minimum distance to HTR.

As shown in Figures 1, 2, we observed no significant differences between the LOA and EOA groups in terms of minimum distance to the nearest HTR (EOA vs. LOA: 1,124 ± 787 m vs. 1,412 ± 175 m; *p* = 0.07). We further analyzed the number of HTRs within regions that were measured from patient residences at set distances of up to 1,000 m. When patients resided with a distance of 900 m in the high-traffic road, more patients with LOA were living in multiple HTRs areas compared to patients with EOA (≥ 2 HTRs; 14.3 vs. 3.4%, *p* = 0.023. Table 2). Subgroup analysis was performed to identify patient characteristics associated with multiple HTRs within 900 m. Briefly, this involved pooling 30 patients with LOA with two patients with EOA for analysis. Compared to patients living in fewer than two HTRs within 900 m, those living in more than two HTRs had higher IgE levels (Table 3, mean ± *SD*, 426.9.1 ± 798.2 vs. 252.9 ± 360.8 KU/L, *p* = 0.05), more positive atopic status (75 vs. 49.8%, *p* = 0.02), and a higher sensitivity to HDM (64.3 vs. 43.2%, *p* = 0.04).

**Table 2:** The density of heavy traffic roads proximal to residences of patients with EOA or LOA^*^.

	**EOA (*n =* 58)**	**LOA (*n =* 210)**	***p*-value**
**Road density within 900 m**			
Heavy traffic roads ≥1	27 (46.6)	100 (47.6)	0.86
Heavy traffic roads ≥2	2 (3.4)	30 (14.3)	0.02
**Road density within 600 m**			
Heavy traffic roads ≥1	16 (27.6)	77 (36.7)	0.19
Heavy traffic roads ≥2	1 (1.7)	20 (9.5)	0.05
**Road density within 300 m**			
Heavy traffic roads ≥1	9 (15.5)	47 (22.4)	0.249
Heavy traffic roads ≥2	0	5 (2.4)	0.234
**Road density within 100 m**			
Heavy traffic roads ≥1	5 (8.6)	17 (8.1)	0.905
Heavy traffic roads ≥2	0	1 (0.5)	0.598

* *Patients living in New Taipei City, Taoyuan, and Hsinchu, Taiwan*.

*Data are presented as patient numbers and percentages*.

**Table 3:** Asthma-associated inflammatory markers in asthmatic patients living in areas with or without high-density traffic as indicated by at least two heavy traffic roads within 900 meters.

	**Residence with**	**Residence with**	***p* value**
	**≥ 2 major roads**	**≤ 1 major road**	
	**(*n =* 32)**	**(*n* = 236)**	
IgE level, KU/L, median (range)	144.0 (6–3,548)	105.0 (0–2,075)	0.05
ECP level, μg/L	16.1 (13.4)	15.0 (25.7)	0.7
Eosinophil counts, cells/μL	170.6 (95.4)	217.0 (226.8)	0.3
Atopy, % (n/N)	75.0 (21/28)	49.8 (100/201)	0.02
**Positive of specific IgE**			
Home dust mite, % (n/N)	64.3 (18/28)	43.2 (83/192)	0.04
Cockroach, % (n/N)	32.1 (9/28)	19.8 (38/192)	0.1
Cat dander, % (n/N)	10.7 (3/28)	13.0 (25/192)	1.0
Dog dander, % (n/N)	17.9 (5/28)	14.1 (27/192)	0.6
Blomia tropicalis, % (n/N)	450.0 (13/26)	40.7 (72/177)	0.4
Penicillium natatum, % (n/N)	17.6 (3/17)	5.6 (9/161)	0.09
Cladosporium herbarum, % (n/N)	11.8 (2/17)	1.9 (3/161)	0.07

*Data are presented as number and percentage, mean and standard deviation (SD), or percentage and positive proportion*.

*N, number of participants who provided information; n, number of participants with positive results*.

*EOA, early-onset asthma; LOA, late-onset asthma; IgE, immunoglobulin E; ECP, eosinophil cationic protein*.

### The Associations of Patients With LOA With the Minimum Distance to HTRs

**Figure 3:**
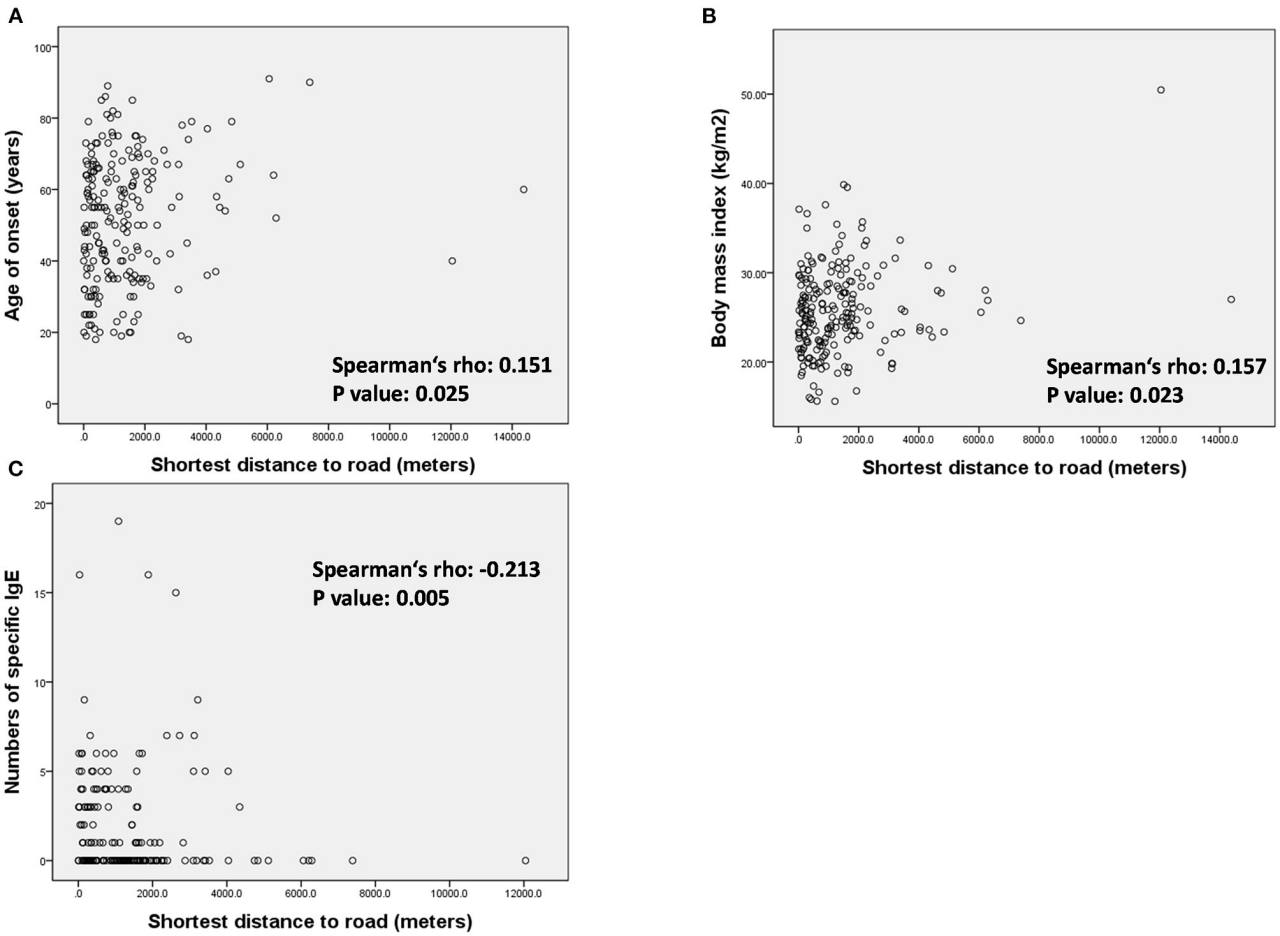
Association between minimum distance to heavy traffic road and **(A)** age of onset, **(B)** body mass index, and **(C)** number of specific IgE in the patients with LOA.

To clarify the influence of high traffic density on patients with LOA, we conducted further analysis on the correlation between the distance to HTRs and patient characteristics in the LOA group (Figure 3 and Supplementary Table S2). The minimum distance to an HTR was positively correlated with age of onset (Figure 3A, Spearman's rho 0.151, *p* = 0.025), BMI (Figure 3B, Spearman's rho 0.157, *p* = 0.023) as well as negatively correlated with the numbers of positive specific IgEs (Figure 3C, Spearman's rho −0.213, *p* = 0.005).

### The Characteristics of Patients With LOA According to the Distance Away From HTRs

**Figure 4:**
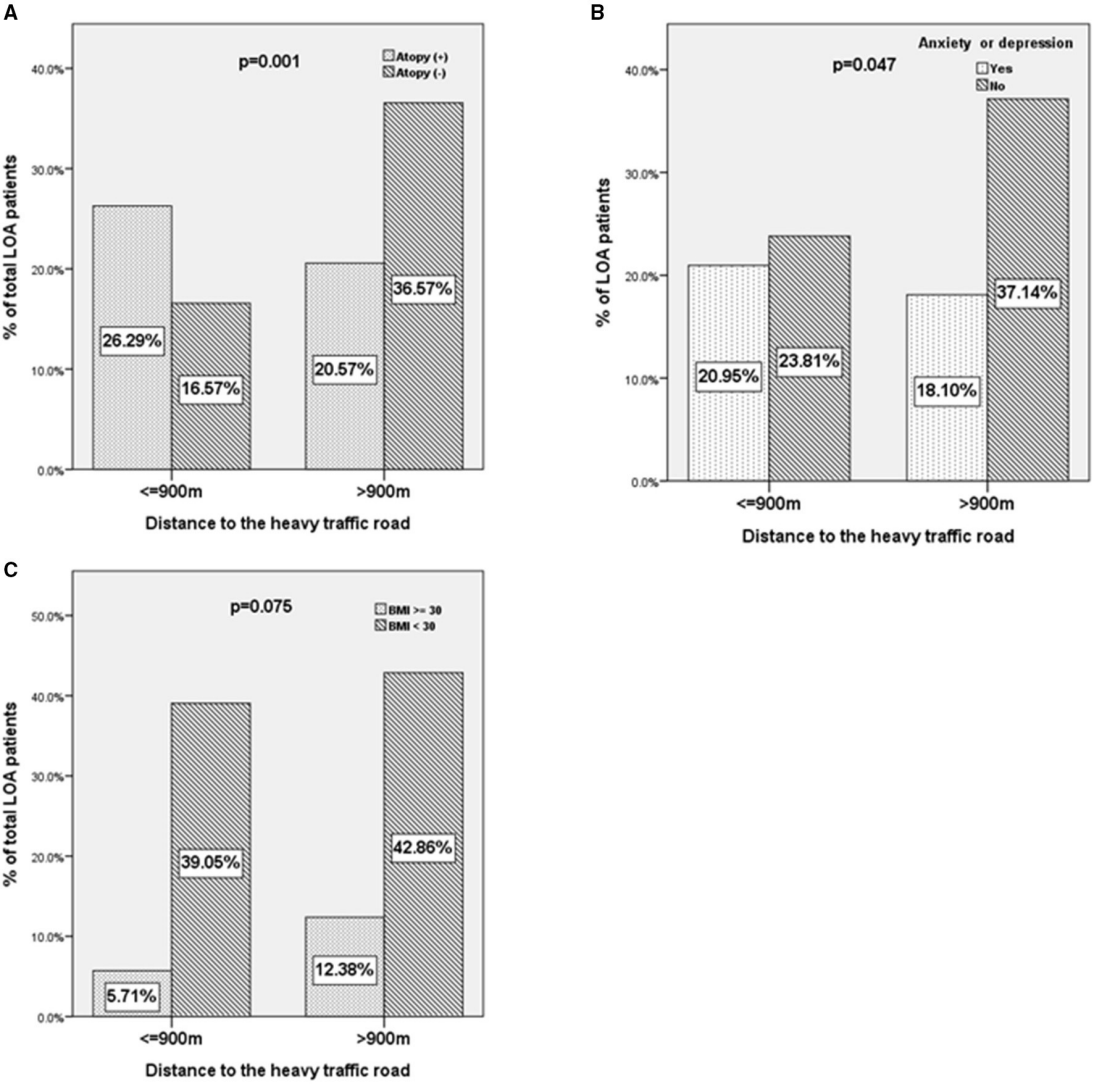
The characteristics of patients with LOA according to the distance away from heavy traffic roads. **(A)** Atopy status, **(B)** anxiety or depression, and **(C)** body mass index ≥30 of patients with LOA as a function of the distance between residence and heavy traffic roads (≤900 vs. >900 m).

For the binary variables and confirming the results of the correlation test, we compared the atopy, mood status, and obesity (BMI ≥ 30) of patients with LOA living within or beyond 900 m of HTRs (Figure 4). A higher proportion of patients with LOA with atopic status (26.3 vs. 20.5%, *p* = 0.001, unadjusted OR: 2.82, 95%CI: 1.519–5.235. Figure 4A) and depression or anxiety were found to be living within 900 m from HTRs (21.0 vs. 18.1%, *p* = 0.047, unadjusted OR: 1.81, 95%CI: 1.031–3.165. Figure 4B). In contrast, there was a trend of a higher proportion of obese patients were living beyond 900 m from HTRs (12.4 vs. 5.7%, *p* = 0.075, unadjusted OR: 1.974, 95%CI: 0.936–4.165. Figure 4C). Interestingly, the proportion of obese patients living beyond 1,000 m from HTRs was statistically higher than those living within 1,000 m to HTRs (data not shown).

## Discussion

Our analysis revealed that patients with LOA tended to be older than patients with EOA, to have had asthma for a shorter duration, were less likely to have a family history of asthma, were less exposed to fumes or dust in the home, were less likely to be atopic, were less sensitive to common inhaled allergens, and were more likely to have poor lung function. In urban areas, more patients with LOA had multiple HTRs (≥ 2 HTRs) within a distance of 900 m. Patients living within 900 m from multiple HTRs had a higher total IgE level, a more atopic status, and a higher sensitivity to HDM, compared to patients with ≥ 1 HTRs at a comparable distance. Among patients with LOA, the minimum distance to an HTR was positively associated with the age of onset and BMI and was negatively associated with atopy and mood status. Among patients with LOA, a higher proportion of atopic patients were living within 900 m from HTRs, a higher proportion of patients with anxiety or depression were living within 900 m from HTRs and a trend of a higher proportion of obese patients were living beyond 900 m from HTRs. To the best of our knowledge, this is the first study demonstrating the different traffic density between EOA and LOA and different phenotypes in LOA by geolocation, which showed the novel relation between asthma phenotypes and the urban environment.

Correlation between TRAP and asthma is usually assessed in terms of pollutant concentration, such as nitrogen oxide and particulate matter (PM), or the distance to HTRs. Numerous cohort studies have demonstrated a positive association between exposure to TRAP and the risk of asthma (11, 23, 29, 30); however, studies on the link between proximity to HTRs and the risk of asthma have been inconsistent. A birth cohort study in southwestern British Columbia failed to observe a significant correlation between proximity to highways and major roads (<150 m) and development of childhood asthma (15). A birth cohort study in New York City reported a significant correlation between proximity to heavy traffic (<250 m) and childhood asthma among patients without a history of moving prior to the age of 5 (16). In a cohort study of children with asthma attending elementary school in an urban area of the northeastern United States, the incidence of asthma symptoms was shown to increase inversely with the distance to major roads (12). One cohort study conducted in three cities in Sweden reported a positive association between adult-onset asthma and proximity (<50 m) to major roads (≥8,000 vehicles/day) (23). Two cross-sectional studies examined the links between subgroups of asthma and proximity to major roads. One study on adults in southern Sweden discovered that proximity (>100 m) to a major road (>10 cars/min) was associated with allergic asthma but not with non-allergic asthma (24). One study on adults in Tasmania, Australia, (i.e., an area with low air pollution levels) reported that proximity (<200 m) to a major road was associated with an elevated risk of asthma; however, this was only among carriers of glutathione S-transferase theta-1 (25). The different distances to HTR for asthma risk in the studies above may result from the different traffic volumes, the population in risks, and the local environment of individual cities. In the current study, HTRs were defined by daily mean PCU exceeding 36,329, which was much higher than that of other studies (23, 24). The range of 1,000 m to HTR was selected initially because the relevant reports included one study from our asthma patients (26, 28). By defining HTRs with the top-ten high traffic volume from the official database, we determined that living within 900 m of multiple HTRs was associated with an elevated risk of LOA compared to EOA. The current study provides new evidence of the greater impact of heavy traffic exposure for LOA. In addition to the direct distance to HTRs, exposure to the density of HTRs is also an important determinant for asthma control.

The patient profiles revealed by the subgroup analysis of patients living with multiple HTRs provided one of the possible mechanisms of asthma inception of LOA (Table 3). Patients living within 900 m from multiple HTRs presented higher total IgE levels, were more likely to be atopic and were more likely to present sensitivity to HDM, compared to patients with fewer than two HTRs at a comparable distance. We also found that asthma onset was earlier among patients with LOA living near HTRs. This suggests that an interaction between TRAP and allergic sensitization may be the force driving asthma inception at an early age among LOA living near Figures 3, 4. Similar to our results, a cross-sectional study revealed associations between exposure to high road density and the prevalence of allergic sensitization and small airway function in subjects with a family history of asthma (31). Previous animal studies on TRAP exposure and allergic sensitization support our results. In adult and neonatal mice, co-exposure to diesel exhaust particles (DEPs) and house dust mites was shown to promote the persistence of TH2/TH17 effector/memory cells in the lungs (32). In studies in an adult mouse model, DEP and HDM co-exposure has also been shown to enhance airway hyper-responsiveness and generate a mixed TH2 and TH17 response or the number of type 2 innate lymphoid cells (33, 34). Co-exposure to HDM and benzo(a)pyrene has also been shown to enhance IL-33 and TSLP production in an asthma mouse model (35). Environmental factors (e.g., ambient air polyaromatic hydrocarbons, PM, and DEP) have been linked to epigenetic changes that modify the gene expression of T regulatory cells or innate response, further promoting the Th2 response (36).

Anxiety and depression contribute to asthma symptoms and stressful life events have been shown as the risk factor of onset of asthma (37, 38). Some studies have shown psychiatric stress enhances allergic inflammation (39, 40). We found the risk of anxiety or depression increased in patients living within 900 m to HTRs (Figure 4B). This finding further demonstrated the complex interactions between the psychiatric status, traffic exposure in patients with LOA, and further studies for better analysis are required.

The incidence of obesity in patients with LOA was higher among those living >900 m from HTRs than among those living <900 m from HTRs (Figure 4C). This conflicts with a number of previous studies suggesting that air pollution plays a role in the incidence of asthma among the obese (41). By contrast, the incidence of atopic asthma in patients with LOA was lower among those living far from HTRs (Figures 3, 4). We surmise that exposure to TRAP may play a more important role in the pathogenesis of atopic asthmatics than it does in obese asthmatics. Future studies on obesity-related LOA and TRAP in urban areas are required.

The study has several limitations. First, the cohort had a smaller patient number of EOA compared to LOA. This is because we recruited adult patients consecutively without selection in the pay-for-performance program to prevent selection bias. The higher ratio of LOA to EOA in our cohort is possible due to the remission rate of EOA being much higher than LOA and LOA is suggested to be more severe than EOA (2, 42). Therefore, similar to other cohorts (43), patients with LOA were referred from local clinics to our center more frequently than patients with EOA. The present results from a single center will have to be confirmed further in subsequent longitudinal studies in a larger population. Second, self-reports pertaining to the age of the patient at the time of diagnosis by a physician were subject to recall bias. Note that we were unable to obtain documented medical records related to asthma diagnosis; however, the patient characteristics in the current study (e.g., family history of lung function) were comparable with those obtained in large-cohort studies and major review articles related to LOA (2, 3, 6, 9, 18, 44). Third, covariates of exposure to fume or dust or comorbidities were defined by questionnaires or medical records, not by real inspection of patients' environments or strict medical diagnostic criteria. The results of a negative association between exposure and comorbidities and HTR proximity in the current study are required further studies to confirm. Fourth, we were unable to obtain information related to TRAP concentrations; therefore, our findings are not necessarily generalizable to all HTRs. Our findings could have been affected by local climatic conditions, occupational exposure, and indoor air pollution in the individual urban environment.

In conclusion, the characteristics of patients with LOA were distinct from those of patients with EOA. By geolocation in urban centers, we discovered the correlation between asthma phenotypes and the urban environment in LOA. patients with LOA tended to live in areas of higher HTR density, which was associated with an elevated incidence of atopic symptoms, sensitivity to HDM, and mood disorder. Proximity to HTRs and obesity may be factors contributing to uncontrolled asthma in cases of LOA. The novel evidence of patient-environment interaction provides further explanation for asthma persistence in the modern world.

## Data Availability Statement

The raw data supporting the conclusions of this article will be made available by the authors, without undue reservation.

## Ethics Statement

The studies involving human participants were reviewed and approved by Chang Gung Medical Foundation Institutional Review Board (No. 201900211B0). The patients/participants provided their written informed consent to participate in this study.

## Author Contributions

T-YL: conceptualization, data curation, investigation, formal analysis, and writing of the original draft. Y-SL: methodology, software, formal analysis, and visualization. H-CL: data curation, resources, and formal analysis. S-ML: data curation, resources supervision, formal analysis, and writing with review and editing. Y-LL, C-HW, P-JC, and C-YL: data curation and resources. All authors contributed to the article and approved the submitted version.

## Conflict of Interest

Y-SL is employed by BalDr Strategic Consulting (Hong Kong) Ltd. The remaining authors declare that the research was conducted in the absence of any commercial or financial relationships that could be construed as a potential conflict of interest.

## Publisher's Note

All claims expressed in this article are solely those of the authors and do not necessarily represent those of their affiliated organizations, or those of the publisher, the editors and the reviewers. Any product that may be evaluated in this article, or claim that may be made by its manufacturer, is not guaranteed or endorsed by the publisher.
