# Supplementary material for: Proximity to Heavy Traffic Roads and Patient Characteristics of Late of Onset Asthma in an Urban Asthma Center
Source: Front Med (Lausanne). 2021 Dec 16;8:783720. doi: 10.3389/fmed.2021.783720 (PMC8716741; doi:10.3389/fmed.2021.783720)
Supplement: Supplementary file 1 [file Data_Sheet_1.doc]

**Figure S1.** Analysis plan in the current study. The entire cohort was first analyzed in terms of patient characteristics and divided into early onset asthma (EOA) and late onset asthma (LOA). The urban dwelling patients (94.7% of the total) were subjected to further analysis based on geometric data.


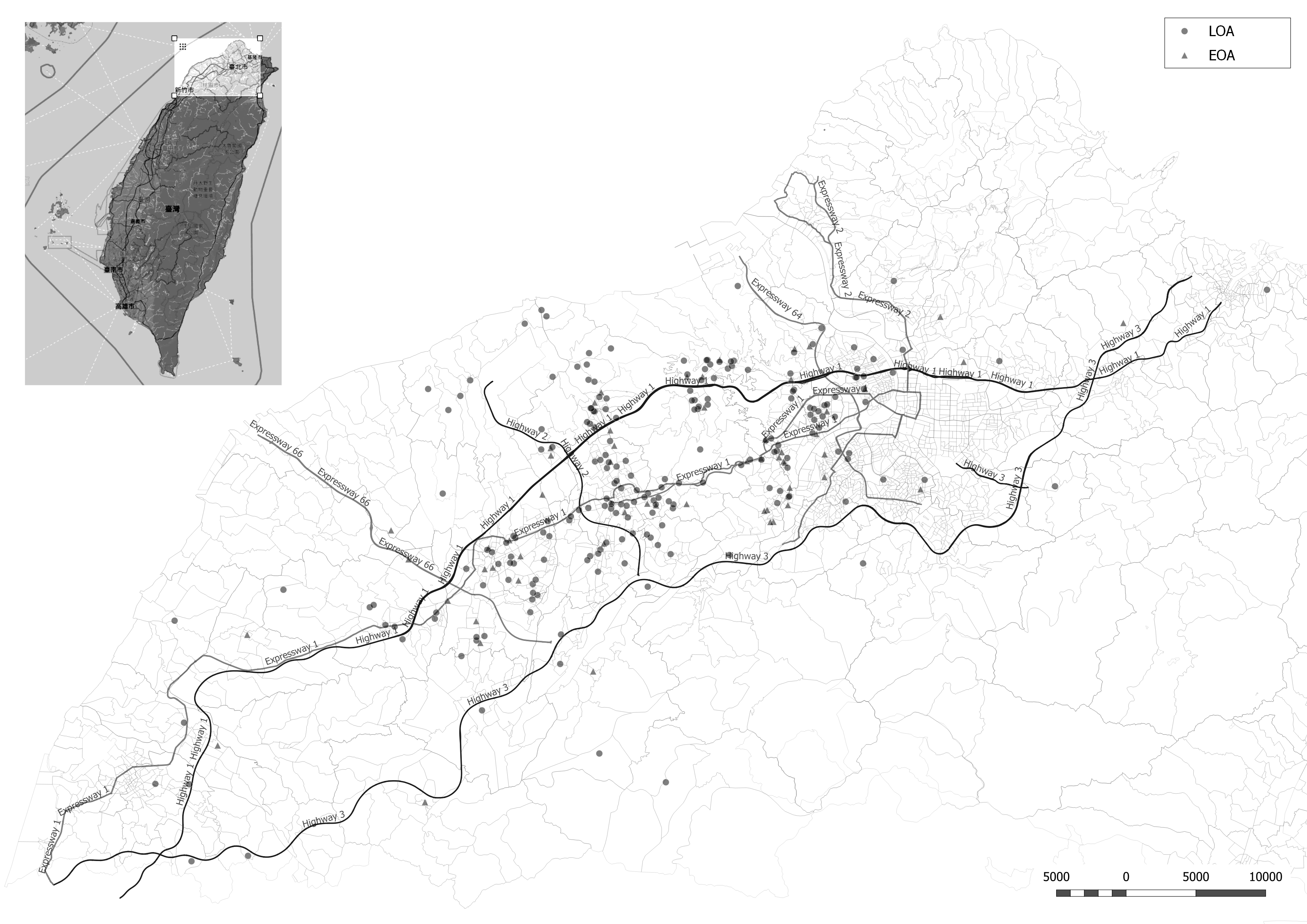


**Figure S2.** Locations indicating the residences of patients with EOA (labeled by triangles) and LOA (labeled by circles) in proximity to three heavy traffic roads: three national highways (Highway 1, 2, and 3) and five expressways (Expressway 1, 2, 3, 64, and 66) in the New Taipei City, Taoyuan, and Hsinchu, in northern Taiwan.


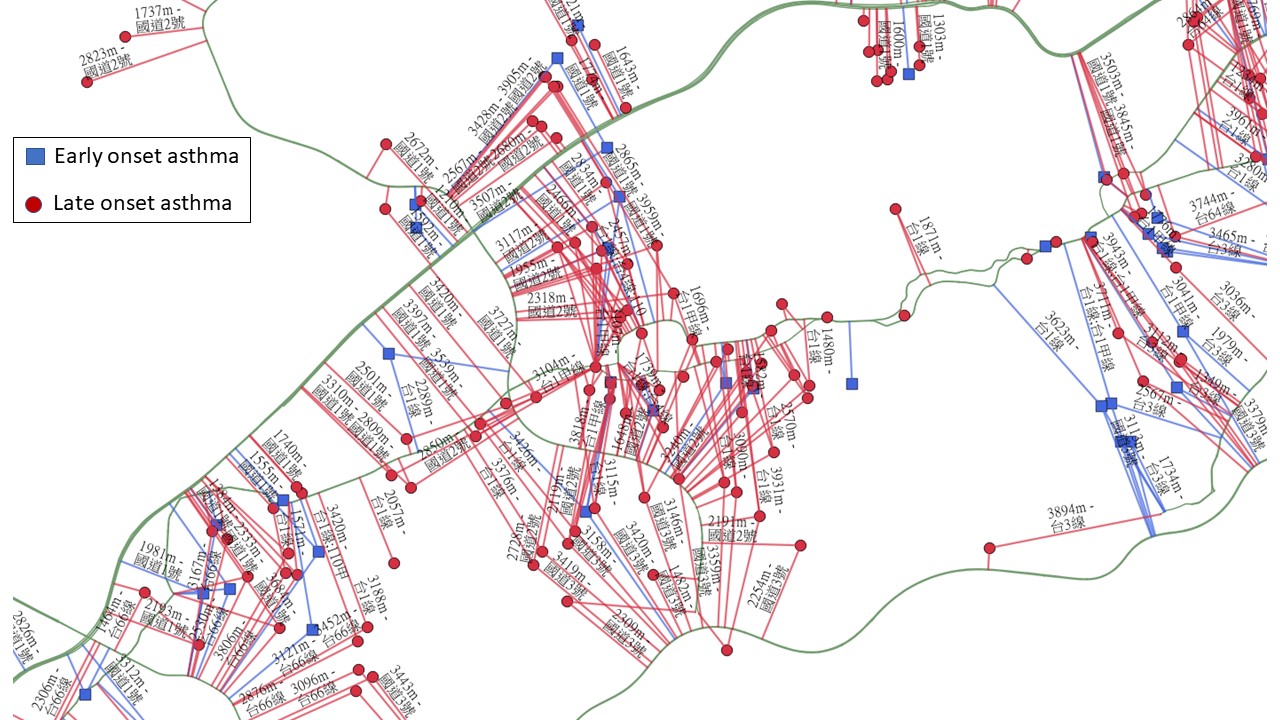


**Figure S3.** Example of the measurement the traffic proximity and density of patients. Linear algebra was used to calculate the minimum distance between the domicile of each patient and the nearest HTR. We also calculated the overall density of traffic in the areas surrounding the domicile of each patient by counting the number of HTRs within the circle of 1 km. The graphical representation of input variables and calculation results was handled using QGIS (v3.10.1) software.


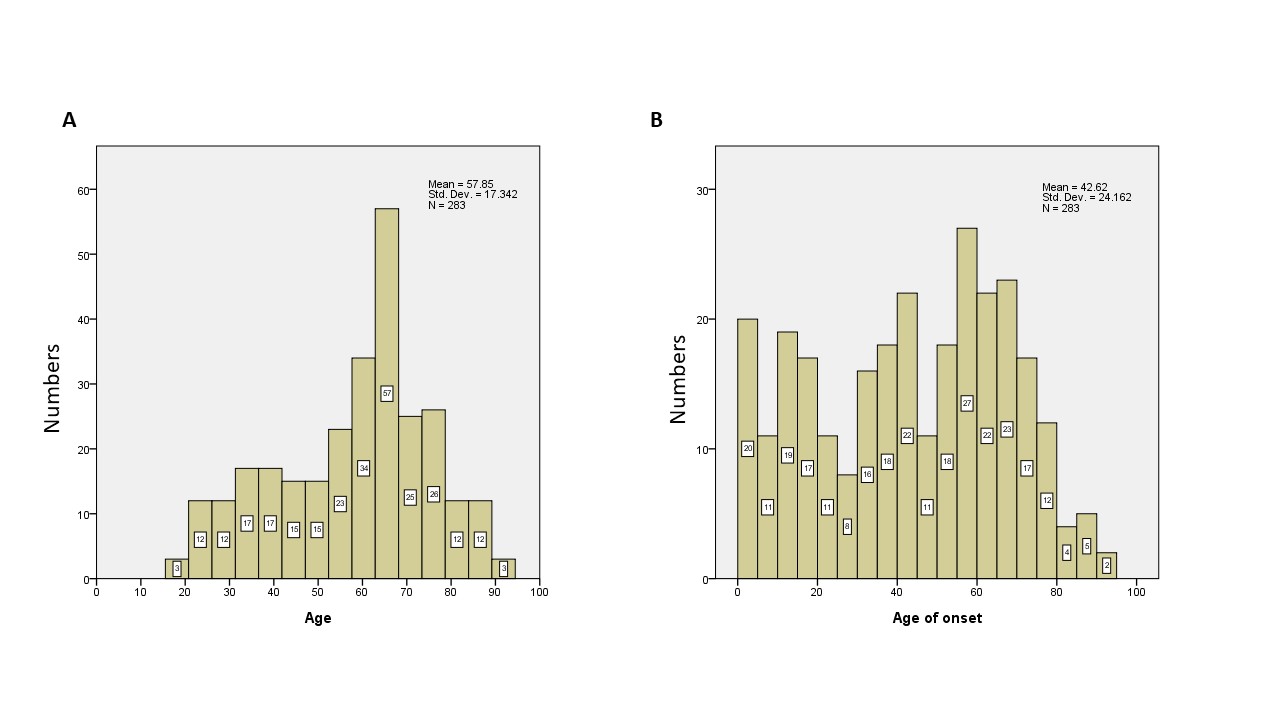


**Figure S4.** A. Age distribution of entire cohort. The mean age with standard deviation was 57.9±17.3 years old. B. Age distribution at time of asthma onset in the entire cohort.

**Table S1. Common specific immunoglobulin E to allergens in EOA and LOA groups**

|  | EOA | LOA | p value |
| --- | --- | --- | --- |
| Home dust mite, % (n/N) | 75.5 (40/53) | 36.5 (64/176) | <0.001 |
| Cockroach, % (n/N) | 24.5 (13/53) | 19.3 (34/176) | 0.4 |
| Cat dander, % (n/N) | 22.6 (12/53) | 9.1 (16/176) | 0.015 |
| Dog dander, % (n/N) | 32.2 (16/53) | 9.1 (16/176) | <0.001 |
| Penicillium natatum, % (n/N) | 4.5 (2/44) | 7.1 (10/141) | 0.7 |
| Cladosporium herbarum, % (n/N) | 2.3 (1/44) | 3.5 (5/141) | 1.0 |
| Aspergillus fumigatus, % (n/N) | 6.8 (3/44) | 4.3 (6/141) | 0.4 |

Data are presented as percentage and positive proportion.

N: number of participants who provided information, n: number of participants with positive result.

Abbreviations: EOA: early-onset asthma; LOA: late-onset asthma

**Table S2. Correlations between minimum distance to heavy traffic roads and patient characteristics in LOA group**

| **Variables** | **Spearman's ρ** | **p value** |
| --- | --- | --- |
| **Patient characteristics** | | |
| Age of onset, years | 0.151 | 0.025 |
| Age, years | 0.107 | 0.123 |
| Asthma duration, years | -0.056 | 0.417 |
| Body mass index, kg/m^2^ | 0.157 | 0.023 |
| ACT score | -0.052 | 0.527 |
| **Asthma-associated inflammatory markers, atopic status** | | |
| IgE level, KU/L | -0.102 | 0.185 |
| Numbers of specific IgE | -0.213 | 0.005 |
| ECP level, μg/L | -0.058 | 0.470 |
| Eosinophil, % | -0.014 | 0.870 |
| Eosinophil counts, cells/μL | -0.080 | 0.989 |
| WBC, cells/uL | -0.039 | 0.629 |
| **Pulmonary function at screening** | | |
| Pre-BD FVC, % of pred. | -0.020 | 0.781 |
| Pre-BD FEV1, % of pred. | -0.008 | 0.907 |
| FEV1/FVC (%) | -0.046 | 0.514 |
| FEV1 % change to BD | -0.004 | 0.954 |

Associations were tested by Spearman’s rank correlation.

Abbreviations: LOA: late-onset asthma; IgE: immunoglobulin E; ECP: eosinophil cationic protein; FVC: forced vital capacity; FEV1: forced expiratory volume in one second; BD: bronchodilator; pred.: prediction; ACT: asthma control test.
